# Pro-inflammatory cytokines stimulate CFTR-dependent anion secretion in pancreatic ductal epithelium

**DOI:** 10.1186/s11658-024-00537-1

**Published:** 2024-01-23

**Authors:** Dora Angyal, Tessa A. Groeneweg, Anny Leung, Max Desain, Kalyan Dulla, Hugo R. de Jonge, Marcel J. C. Bijvelds

**Affiliations:** 1 Department of Gastroenterology and Hepatology, Erasmus MC University Medical Center, PO Box 2040, 3000CA Rotterdam, The Netherlands; 2 Boehringer Ingelheim Pharma GmbH & Co. KG, Binger Strasse 173, 55216 Ingelheim Am Rhein, Germany

**Keywords:** CFTR, Cystic fibrosis, Cytokines, Epithelial ion transport, Organoid, Pancreatitis

## Abstract

**Background:**

Loss of CFTR-dependent anion and fluid secretion in the ducts of the exocrine pancreas is thought to contribute to the development of pancreatitis, but little is known about the impact of inflammation on ductal CFTR function. Here we used adult stem cell-derived cell cultures (organoids) obtained from porcine pancreas to evaluate the effects of pro-inflammatory cytokines on CFTR function.

**Methods:**

Organoids were cultured from porcine pancreas and used to prepare ductal epithelial monolayers. Monolayers were characterized by immunocytochemistry. Epithelial bicarbonate and chloride secretion, and the effect of IL-1β, IL-6, IFN-γ, and TNF-α on CFTR function was assessed by electrophysiology.

**Results:**

Immunolocalization of ductal markers, including CFTR, keratin 7, and zonula occludens 1, demonstrated that organoid-derived cells formed a highly polarized epithelium. Stimulation by secretin or VIP triggered CFTR-dependent anion secretion across epithelial monolayers, whereas purinergic receptor stimulation by UTP, elicited CFTR-independent anion secretion. Most of the anion secretory response was attributable to bicarbonate transport. The combination of IL-1β, IL-6, IFN-γ, and TNF-α markedly enhanced CFTR expression and anion secretion across ductal epithelial monolayers, whereas these cytokines had little effect when tested separately. Although TNF-α triggered apoptotic signaling, epithelial barrier function was not significantly affected by cytokine exposure.

**Conclusions:**

Pro-inflammatory cytokines enhance CFTR-dependent anion secretion across pancreatic ductal epithelium. We propose that up-regulation of CFTR in the early stages of the inflammatory response, may serve to promote the removal of pathogenic stimuli from the ductal tree, and limit tissue injury.

**Supplementary Information:**

The online version contains supplementary material available at 10.1186/s11658-024-00537-1.

## Introduction

Acute pancreatitis is an inflammatory disease of the exocrine pancreas, which may develop under sterile conditions and is thought to be instigated by acinar cell injury [1, 2]. Apart from transient obstruction of the pancreatic ducts by gallstones, which causes bile to enter the pancreatic ductal tree, the most common causes of acute pancreatitis are alcohol abuse and hypertriglyceridemia [2, 3]. The inflammatory response may resolve without specific treatment within a few days, or progress to systemic inflammation, culminating in a life-threatening failure of organs outside the pancreas [2]. Acute pancreatitis is relatively prevalent, accounting for a substantial portion of the hospitalizations that are related to the gastrointestinal tract [2, 4]. Treatment relies principally on supportive care.

The digestive enzyme-producing acinar cells of the exocrine pancreas seem to be especially susceptible to pathogenic stimuli. Typical stressors associated with pancreatitis, like ethanol or bile acids are thought to disrupt Ca^2+^ homeostasis in these cells, leading to the untimely activation of proteolytic enzymes, principally trypsin, and cell death [1–4]. Pathogenic stimuli also promote the activity of NFκB in acinar cells, which, together with the release of damage-associated molecular patterns (DAMPs) by injured cells, leads to the activation of stellate cells and immune cell infiltration [3–5]. The release of pro-inflammatory cytokines, including tumor necrosis factor-α (TNF-α), interleukin-1β (IL-1β) and interleukin-6 (IL-6), by antigen presenting cells (dendritic cells, macrophages), and interferon γ (IFN-γ) by Th1 cells, causes further tissue damage, and may also promote the translocation of bacteria across the intestinal mucosa, leading to exposure of the pancreas to further pro-inflammatory stimuli [3–6].

Multiple genetic factors influence the susceptibility to environmental triggers of pancreatitis [7–9]. One such factor is the *CFTR* (cystic fibrosis transmembrane conductance regulator) gene, which encodes a phosphorylation-regulated chloride/bicarbonate channel, principally expressed in epithelia of the respiratory and gastrointestinal tract. Loss-of-function mutations in *CFTR* cause cystic fibrosis (CF), a complex, multi-organ disease, with potentially fatal outcome [10]. In the pancreatic ductal epithelium, CFTR-mediated anion transport is essential for the formation of an alkaline, bicarbonate-rich fluid [10, 11]. In the majority of CF patients, i.e. those carrying *CFTR* alleles with little or no residual function, obstruction of the ducts by mucus and protein aggregates leads to atrophy and fibrosis, and exocrine pancreatic insufficiency develops early in life [10]. Importantly, some more benign mutations in the CFTR gene, while not linked to early onset CF-typical pancreatic fibrosis and insufficiency, seem to predispose to the development of pancreatitis, and it has been estimated that circa 30% of individuals diagnosed with idiopathic pancreatitis carry mutations in *CFTR* [12–16]. Pancreatitis is also a typical manifestation of CFTR-related disease, a range of disorders not meeting criteria for CF diagnosis, but with evidence for moderate CFTR dysfunction, and CF-like pathology often presenting in a single organ [17]. Collectively, these findings suggest that the pancreas is eminently sensitive to impairment of CFTR function, and that even a subtle reduction in CFTR activity renders the pancreas more vulnerable to an environmental stressor or a mutation in another gene associated with pancreatitis, i.e. a second hit [1, 2, 7, 12, 15, 18].

Consistent with the concept that ductal CFTR has an essential tissue-protective role in the pancreas by containing the risk of tissue damage upon exposure to pathogenic stimuli, it was proposed that a reduction in ductal fluid secretion initiates and/or propagates some acute forms of pancreatitis. Typical stressors associated with pancreatitis, like trypsin, bile acids, ethanol and palmitoleic acid (which may be elevated in hypertriglyceridemia) were all shown to acutely reduce anion secretion in pancreatic ducts [18–21]. However, little is known about the initial effects of inflammatory mediators released by infiltrating immune cells on CFTR activity in the pancreatic ductal epithelium. This question is pertinent, in view of the fact that several cytokines were shown to regulate the activity of CFTR and other anion transport mechanisms in epithelial tissues. For instance, it was reported that IL-1β and TNF-α stimulate CFTR-mediated fluid secretion in the submucosal glands of the respiratory tract, and that IL-1β induces expression of CFTR in human airway epithelium-derived Calu-3 cells [22, 23]. Further, interleukin 4 (IL-4) was shown to stimulate CFTR-mediated chloride secretion across airway surface epithelia via a pendrin (*SLC26A4*)-dependent mechanism [24]. Similarly, the combination of TNF-α and interleukin 17 (IL-17) was reported to strongly induce *SLC26A4* in airway epithelium, and to alkalinize airway surface liquid through stimulation of CFTR-dependent bicarbonate secretion [25]. Cytokine-dependent alkalization of airway surface liquid, by stimulating mucociliary clearance and killing of bacteria, is thought to serve as a host-protective mechanism [26]. Conversely, in intestinal and biliary epithelial cells, some of the same cytokines seem to repress CFTR activity, indicating that the effects of cytokines differ between tissues [27–29].

In the present study, we sought to further delineate the impact of inflammation on anion secretion across the pancreatic ductal epithelium. For this purpose, we cultured epithelial monolayers from porcine ductal organoids to quantitatively assess CFTR activity in pancreatic ductal epithelium. As in humans, the concentration of bicarbonate in the pancreatic juice produced by pigs is exceptionally high (> 130 mmol/L), and porcine ductal anion and water secretion is highly dependent on CFTR [11, 30, 31]. The latter is poignantly illustrated by the observation that CFTR deficient piglets display severe exocrine pancreatic destruction, starting from an early stage in life, i.e. a phenotype that closely resembles human CF pathology [32, 33]. We found that monolayers derived from primary cultures of ductal cells form a highly polarized epithelium, that recapitulates many structural and functional aspects of the native ductal epithelium. We used this model to characterize ductal anion transport and to assess the effect of cytokines typically associated with pancreatitis on CFTR function.

## Materials and methods

### Animals

For culture of organoids, tissue was obtained from crossbred Yorkshire-Landrace pigs (*Sus scrofa*), aged 10–16 weeks. Pigs were anesthetized and euthanized as published previously [34]. After excision, pancreatic tissue was briefly rinsed with and subsequently stored in ice-cold Ad-DF+++ medium: advanced DMEM/F12 (Gibco), HEPES (10 mmol/L, Gibco), Glutamax (2 mmol/L, Gibco) and Primocin (100 mg/L, InvivoGen). Studies were approved by the Ethical Committee for Animal Experiments of the Erasmus MC (AVD1010020173286).

### Culture of pancreatic ductal organoids and epithelial monolayers

Tissue, collected from the head region of the pancreas, was minced into small fragments, circa 8 mm^3^ in size, and tissue was further dissociated by treatment with collagenase, according to established protocols [35]. After dissociation, ductal structures were selected using a bright-field microscope (Zeiss Primovert), and embedded in extracellular matrix (Matrigel, Corning). Tissue was incubated in Ad-DF+++ containing the growth factors Wnt3a, Noggin, R-Spondin 1, FGF10 and EGF, and PGE_2_ and forskolin [35]. To stimulate organoid development, Y-27632 (10 μmol/L; R&D Systems) was added during the first two days after seeding and passaging. Pancreatic ductal organoids could be expanded for up to 12 generations/passages.

For culture of epithelial monolayers, extracellular matrix-embedded, pancreatic organoids were enzymatically dissociated in TrypLE Express (Thermo Fisher). Because the rate of expansion of organoid lines generally decreased at later passages, we only used organoids at early passages (3–6) for preparing monolayers. After enzyme activity was quenched by addition of ice-cold Ad-DF+++, cells were filtered through a cell strainer (70 µm; Falcon), collected by centrifugation, and suspended in complete organoid culture medium, supplemented with Y-27632. Cells were seeded (6 × 10^5^/cm^2^) either in 96-well plates (#3596, Corning) or on permeable inserts (Transwell #3470; Corning) that had been pretreated with Matrigel diluted in phosphate buffered saline (PBS; 1:20; 0.2 mL/cm^2^) for 2 h at 37 °C. Culture medium was as for extracellular matrix-embedded organoids, except that, to suppress CFTR activity, the adenylyl cyclase agonist forskolin was removed from the medium one day prior to ion transport measurements. Cells were cultured until a confluent monolayer was obtained (10–14 days). In some instances, prior to assessment of anion transport, confluent monolayers were exposed to TNF-α (50 ng/mL; Peprotech), IFN-γ (200 ng/mL, Peprotech), IL-1β (10 ng/mL; Peprotech) and/or IL-6 (100 ng/mL; Peprotech) for 20 h.

### Cell culture

The human colonic adenocarcinoma cell line HT29-CL19A (#T8212; Applied Biological Materials) was cultured in DMEM (Gibco), supplemented with fetal bovine serum (10%; Gibco), penicillin (100 U/mL; Gibco) and streptomycin (100 µg/mL; Gibco). Cell lysates were prepared in NaCl (150 mmol/L), Tris/HCl pH 7.6 (25 mmol/L), Triton X100 (1%), sodium deoxycholate (1%), sodium dodecyl sulfate (0.1%), supplemented with a protease inhibitor cocktail (Roche). Prior to Western blot analysis, cell lysates were stored at − 80 °C.

### Gene expression

Organoids were collected in ice-cold PBS and extracellular matrix was removed by washing twice. RNA was extracted from organoids and organoid-derived epithelial monolayers using the NucleoSpin RNA kit (Macherey–Nagel), and synthesis of cDNA was performed using PrimeScript RT Enzyme Master Mix (Takara Bio Inc.). Quantitative reverse transcriptase PCR (primer sequences shown in Table 1) was performed in SYBR Green I Master mix (Thermo Fisher Scientific) using the StepOnePlus RT-PCR platform (Applied Biosystems, Thermo Fisher Scientific). Median values of assays performed in triplicate were used to determine gene expression levels relative to *YWHAZ*.

**Table 1:** Primer sequences used for RT-PCR

Gene	Accession number	Forward	Reverse
ANO1 (TMEM16A)	XM_021082670.1	CACCTTCATGGAGCACTGGA	GGATGGTCCTCCTCCTCCTC
ANO6 (TMEM16F)	XM_005655713.3	CGCCAGGCAGAACTTGAGTA	CACGTGAGTACACTGTGCCT
CFTR	NM_001104950.1	TCATGCCGGGCACCATTAAA	CGCTTTGATGACACTCCTGTATCTA
SLC4A2 (AE2)	XM_021078861.1	CCGGTTGATGATGTCGAGGT	CAGCTGGTCCCTGTGAAGTC
SLC4A4 (NBCe1)	NM_001030533.1	TCTTTGCGACAGGTCGTGTA	CTTCCACGCTCTCAGTGGAC
SLC9A1 (NHE1)	NM_001007103.1	CCCCCAAGGACCAGTTCATC	AGGTCTACCAGAGGCCGAAT
SLC12A2 (NKCC1)	XM_003123899.5	GGATGGCAAGACTCCAACTCA	CCTCCATCATCAAAAAGCCACC
SLC26A4 (Pendrin)	XM_003357511.4	TGGTGGGATCTGTTGTTCTGA	ACTCTTGCTGCGTCTCTAGC
SLC26A6 (PAT1)	NM_001012298.2	ATGGGGCTGTCAGAAGCG	GCCTCCTCAGCTCCATTGTT
YWHAZ	NM_001315726.1	GGAGCCCGTAGGTCATCTTG	ATTCTCGAGCCATCTGCTGT

For transcriptome analysis, library construction and sequencing was performed at the Beijing Genomics Institute (BGI). Data were processed with CLC genomic workbench 20 (Qiagen) and sequence reads were mapped to the *Sus scrofa* genome data set 11 (NCBI), using default parameters. Data depict reads per 1000 base pair transcript per million reads mapped (RPKM). Datasets are available through NCBI-GEO repository GSE233579.

### Cytochemistry and immunocytochemistry

Pancreatic tissue or epithelial monolayers were fixed in paraformaldehyde (4%) and embedded in paraffin. Hematoxylin–eosin (HE) staining was performed on 5 µm sections, following established protocols. Immuno-detection of ductal proteins was performed using antibodies directed against keratin 7 (M7018, 1:100; Agilent Dako;), SOX9 (AMAb90795, 1:500; Atlas Antibodies), e-cadherin (#3195, 1:300; Cell Signaling Technology), zonula occludens 1 (21773-1-AP, 1:2000; Proteintech) or an affinity-purified polyclonal antibody directed against human CFTR (G449, 1:100), kindly provided by Dr. A.C. Nairn (Yale School of Medicine, New Haven) [36, 37]. Sections were incubated with an appropriate *fluorescently*-tagged secondary antibody, i.e. goat-anti-mouse IgG (A-11032, Invitrogen) or goat-anti-rabbit IgG (A-11008, Invitrogen), and mounted in Vectashield mounting medium supplemented with 4′,6-diamidino-2-phenylindole (DAPI) to stain nuclei (Vector Laboratories). Fluorescence was visualized on a Stellaris 5 low incidence angle upright confocal microscope with a HC PL APO CS2 40x/1.30 oil objective (Leica Microsystems).

### Western blot analysis

To assess cAMP-dependent protein kinase (PKA)-mediated phosphorylation of the vasodilator-stimulated phosphoprotein (VASP), organoids (9 days after seeding) were incubated for 30 min with vasoactive intestinal peptide (VIP; 50 nmol/L; Bachem), secretin (50 nmol/L; Bachem), or forskolin (10 µmol/L) in Meyler solution (mmol/L): 128 NaCl, 4.7 KCl, 1.3 CaCl_2_, 1.0 MgCl_2_, 20 NaHCO_3_, 0.4 NaH_2_PO_4_, 0.3 Na_2_HPO_4_, 10 HEPES, 10 glucose, at 37 °C. Meyler solution was pre-equilibrated at pH 7.3, in a 5% CO_2_ atmosphere. Subsequently, organoids were collected in ice-cold PBS supplemented with NaF (1 mmol/L). After centrifugation (300 × g, 5 min), collected organoids were lysed in NaCl (150 mmol/L), Tris/HCl pH 7.6 (25 mmol/L), Triton X100 (1%), sodium deoxycholate (1%), sodium dodecyl sulfate (0.1%), NaF (5 mmol/L), Na_3_VO_4_ (3 mmol/L), supplemented with a protease inhibitor cocktail (Roche). Lysates were subjected to SDS-PAGE, and proteins were transferred to nitrocellulose membrane. VASP and Ser-157-phosphorylated VASP (pVASP) was detected using antibodies #3132 and #3111 (1:1000; Cell Signaling Technology), respectively. For detection of CFTR, a combination of 3 monoclonal antibodies was used (#432, #450, #596; 1:3000 each), kindly provided by Dr. J. Riordan (University of North Carolina, Chapel Hill) and the Cystic Fibrosis Foundation, through the CFTR antibody distribution program (https://cftrantibodies.web.unc.edu/). Activated, cleaved caspase-3 was detected using antibody #9661 (1:1000, Cell Signaling Technology). Detection of β-actin (Sc-47778, 1:5000; Santa Cruz) or e-cadherin (#3195; 1:1500; Cell Signaling Technology) served as a loading control. Primary antibodies were detected using an appropriate fluorescently-tagged secondary antibody (goat-anti-mouse IgG or goat anti-rabbit IgG; Invitrogen), and fluorescence was detected on an Odyssey imaging system (Licor Biosciences).

### Electrophysiological assessment of epithelial anion transport

Monolayers were mounted in P2302T/P2300-type Ussing chambers (Physiologic Instruments, San Diego), and bathed in either of two solutions. Firstly, combined chloride and bicarbonate transport was assessed in Meyler solution. Secondly, for assessing bicarbonate transport, NaCl and KCl were replaced by the isethionate salts, and CaCl_2_ and MgCl_2_ by the acetic acid salts of these cations. Solutions were maintained at 37 °C, and adjusted to pH 7.3 by continuous gassing with 95% O_2_, 5% CO_2_. For some experiments, a chloride free buffer was prepared as above, except that the bicarbonate concentration was set at 100 mmol/L by isosmotic substitution of sodium-isethionate for NaHCO_3_ (mmol/L: 48 Na-isethionate, 4.7 K-isethionate, 1.3 Ca(CH_3_COO)_2_, 1.0 Mg(CH_3_COO)_2_, 100 NaHCO_3_, 0.4 NaH_2_PO_4_, 0.3 Na_2_HPO_4_, 10 HEPES, 10 glucose). The pH of this solution was adjusted to 8.0 by gassing with 95% O_2_, 5% CO_2_. The osmolality of buffer solutions was routinely checked using a cryoscopic osmometer, and ranged between 294 and 305 mOsm/kg (Osmomat 30, Gonotec GmbH). The transepithelial potential difference across epithelial monolayers was clamped at 0 mV with a VVC-MC8 module (Physiologic Instruments), and the resulting short-circuit current (Isc) was recorded using a PowerLab 8/35 AD-converter and associated software (LabChart 8; AD Instruments).

CFTR activity was stimulated by addition of either secretin (50 nmol/L; Bachem), VIP (50 nmol/L; Bachem), or forskolin (10 µmol/L). Isc responses (∆Isc) shown represent the difference between the maximum Isc recorded after stimulation, and the Isc just prior to stimulation. PPQ-102 (20 µmol/L; Tocris) and bumetanide (50 µmol/L; Sigma-Aldrich) were used to inhibit CFTR or NKCC1 activity, respectively. UTP (50 µmol/L; Sigma-Aldrich) was added to the luminal bath to stimulate purinergic receptors that couple to Ca^2+^-dependent chloride channels (CaCC). UTP responses were assessed in the presence of PPQ-102.

### Caspase-3 and -7 activity

Confluent monolayers in 96-wells plates were exposed to TNF-α (50 ng/mL; Peprotech), IFN-γ (200 ng/mL, Peprotech), IL-1β (10 ng/mL; Peprotech) and/or IL-6 (100 ng/mL; Peprotech) for 20 h. Experiments were performed in culture medium in a 5% CO_2_ atmosphere at 37 °C. CAS3/7 activity was assessed using a commercially available assay kit (SensoLyte; AS-71141, AnaSpec). Fluorescence was measured over a 30 min period using a CytoFluor 4000 microplate reader (λ_ex_ 485/20 and λ_em_ 530/25 nm; Applied Biosystems). Assays were performed in triplicate, and caspase 3/7 activity was determined from the initial reaction velocity, i.e. the increase in relative fluorescence units (RFU)/min, as per instruction of the kit manufacturer. Values presented depict caspase 3/7 activity relative to a paired solvent control.

### Epithelial permeability

To assess the effect of cytokines on epithelial barrier function, fluorescein isothiocyanate (FITC)–dextran (average molecular weight 4 kDa; 100 µg/ml; Sigma-Aldrich) was added to the apical compartment of monolayers cultured on Transwell inserts, in the presence or absence of a combination of cytokines (50 ng/mL TNF-α, 200 ng/mL IFN-γ, 10 ng/mL IL-1β, 100 ng/mL IL-6). After a 20 h incubation period, FITC-dextran accrued in the basolateral compartment was measured using a CytoFluor 4000 microplate reader (λ_ex_ 485/20 and λ_em_ 530/25 nm). For assessing transepithelial electrical resistance (TEER), epithelial monolayers were mounted in Ussing chambers, bathed in Meyler solution, and the transepithelial potential difference was clamped at 0 mV, as described above. The clamping voltage was briefly (2 s) increased from 0 to 5 mV (∆V), and the resulting change in Isc (∆Isc) was monitored to calculate TEER using Ohm’s law (R = ∆V/∆Isc).

### Statistical analysis

Data are presented as means ± standard deviation. Differences between means were statistically analyzed by Student’s t-test (two-sided) or ANOVA, using the Dunnett correction to control for multiple comparisons (Prism 9; Graphpad software).

## Results

### Organoids and epithelial monolayers cultured from porcine pancreas have characteristics typical of ductal epithelium

**Fig. 1:**
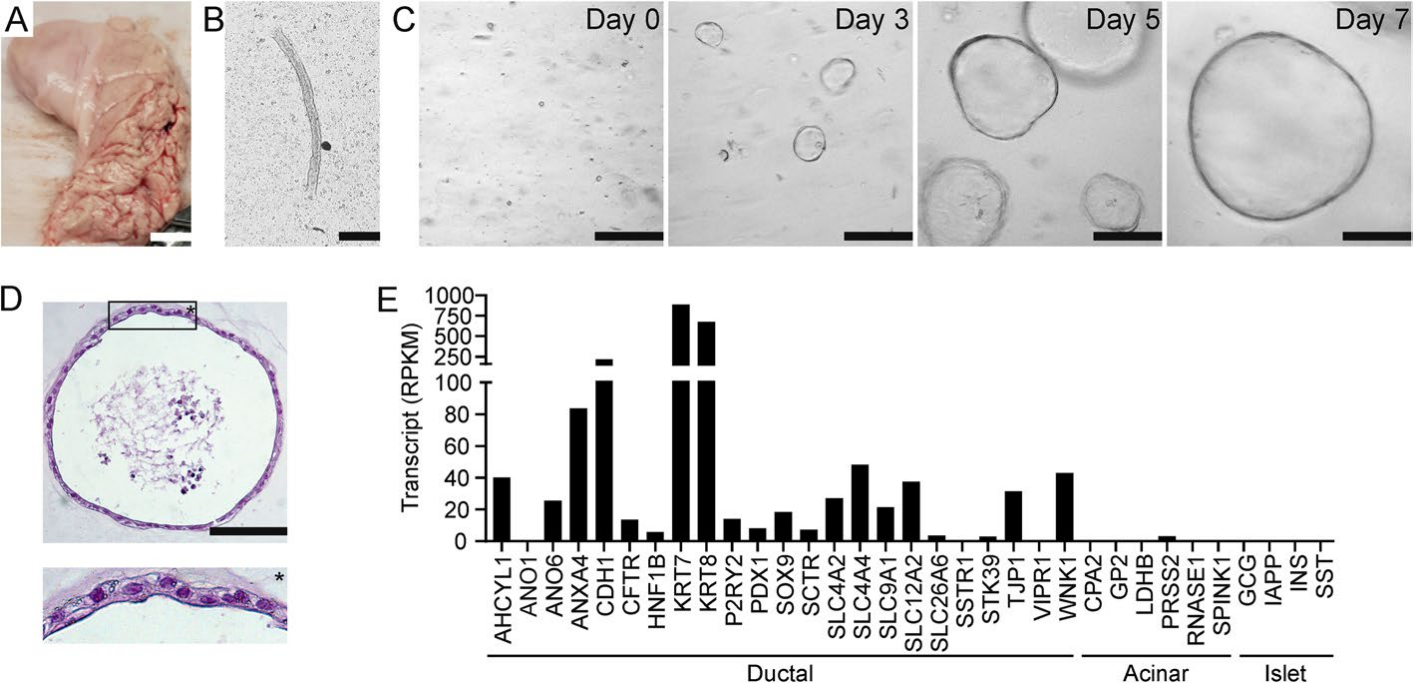
Establishment of pancreatic ductal organoids. **A** Excised pancreatic tissue. Scale bar: 1 cm. **B** Isolated duct (bright-field microscopy). Scale bar: 100 µm. **C** Development of spheroid organoids over the first seven days after seeding. (bright-field microscopy). Scale bars: 1 mm. **D** A pancreatic organoid (HE stain). Scale bar: 100 µm. **E** Transcript levels of ductal, acinar and islet cell markers

Isolated sections of ductal epithelium formed spheroidal structures when cultured in an extracellular matrix. Spheroids consisted of a uniform, single layer of columnar epithelial cells surrounding an apparently fluid-filled central lumen, in keeping with a ductal origin (Fig. 1A–D). Transcriptome analysis indicated that organoid cells express many genes which are characteristic of the pancreatic ductal epithelium, including structural proteins like keratin 7 and 8 (*KRT7*, *KRT8*), e-cadherin (*CDH1*), annexin A4 (*ANXA4*) and zonula occludens 1 (*TJP1*), and transcription factors (*SOX9, HNF1B*; Fig. 1E). Crucially, we also detected transcripts encoding proteins involved in ductal anion secretion. We observed robust expression of the secretin receptor (*SCTR*) as well as the purinergic P2Y2 receptor (*P2RY2*), but comparatively low transcript levels of the type 1 VIP receptor (*VIPR1*) and the somatostatin receptor (*SSTR1*). Cells expressed the principle cellular bicarbonate and chloride uptake mechanisms, i.e. the sodium-coupled cotransporters NBCe1 (*SLC4A4*) and NKCC1 (*SLC12A2*), respectively, the chloride-bicarbonate exchanger AE2 (*SLC4A2*), and the sodium-proton exchanger NHE1 (*SLC9A1*) which, through proton extrusion, contributes to intracellular alkalization [38]. Further, we detected expression of *CFTR* and the chloride-bicarbonate exchanger PAT1 (*SLC26A6*), both key anion transport mechanisms involved in ductal bicarbonate secretion [38]. Cells also expressed the WNK1 and SPAK (*STK39*) protein kinases and IRBIT (inositol 1,4,5-triphosphate (IP_3_) receptors binding protein released with IP_3_; *AHCYL1*), thought to be involved in the regulation of ductal bicarbonate secretion [39, 40]. Interestingly, we observed high transcript levels of anoctamin 6/TMEM16F (*ANO6*), a protein that translocates phospholipids between the inner and outer leaflet of the plasma membrane, but, more tentative, may also function as a Ca^2+^-dependent chloride channel (CaCC) [39, 41]. In contrast, the expression of anoctamin 1/TMEM16A (*ANO1*), a bona fide epithelial CaCC, was comparatively low. Finally, transcript levels of genes typically expressed in pancreatic acinar or islet cells were generally low [42].

**Fig. 2:**
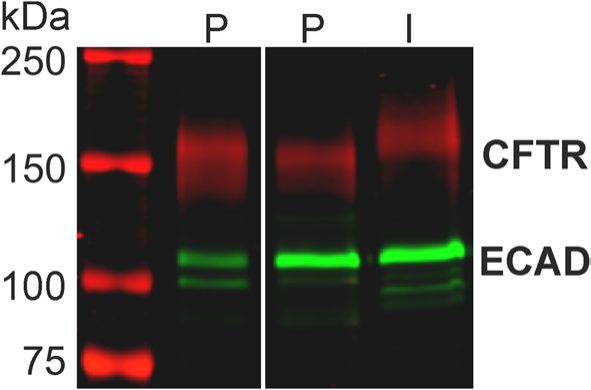
Detection of CFTR protein by Western blot analysis in two pancreatic organoid lines (P) and the human intestinal cell line HT29-CL19A (I). Detection of e-cadherin (ECAD) served as a loading control. Outer left lane shows the position of molecular weight markers, with an approximate molecular mass as indicated

The presence of CFTR protein in ductal organoids was confirmed by Western blot analysis (Fig. 2, Additional file 1: Fig. S1). Using a combination of three monoclonal antibodies, a diffuse immunoreactive band was detected in cell lysates, distinctive of the mature, fully glycosylated protein (*band C*), with an estimated molecular mass of circa 160 kDa. For comparison, CFTR was also detected in HT29-CL19A cells, a human intestinal cell line frequently used to study CFTR function. In line with previous studies we found that the electrophoretic mobility of porcine CFTR is slightly higher than that of the human orthologue [33].

**Fig. 3:**
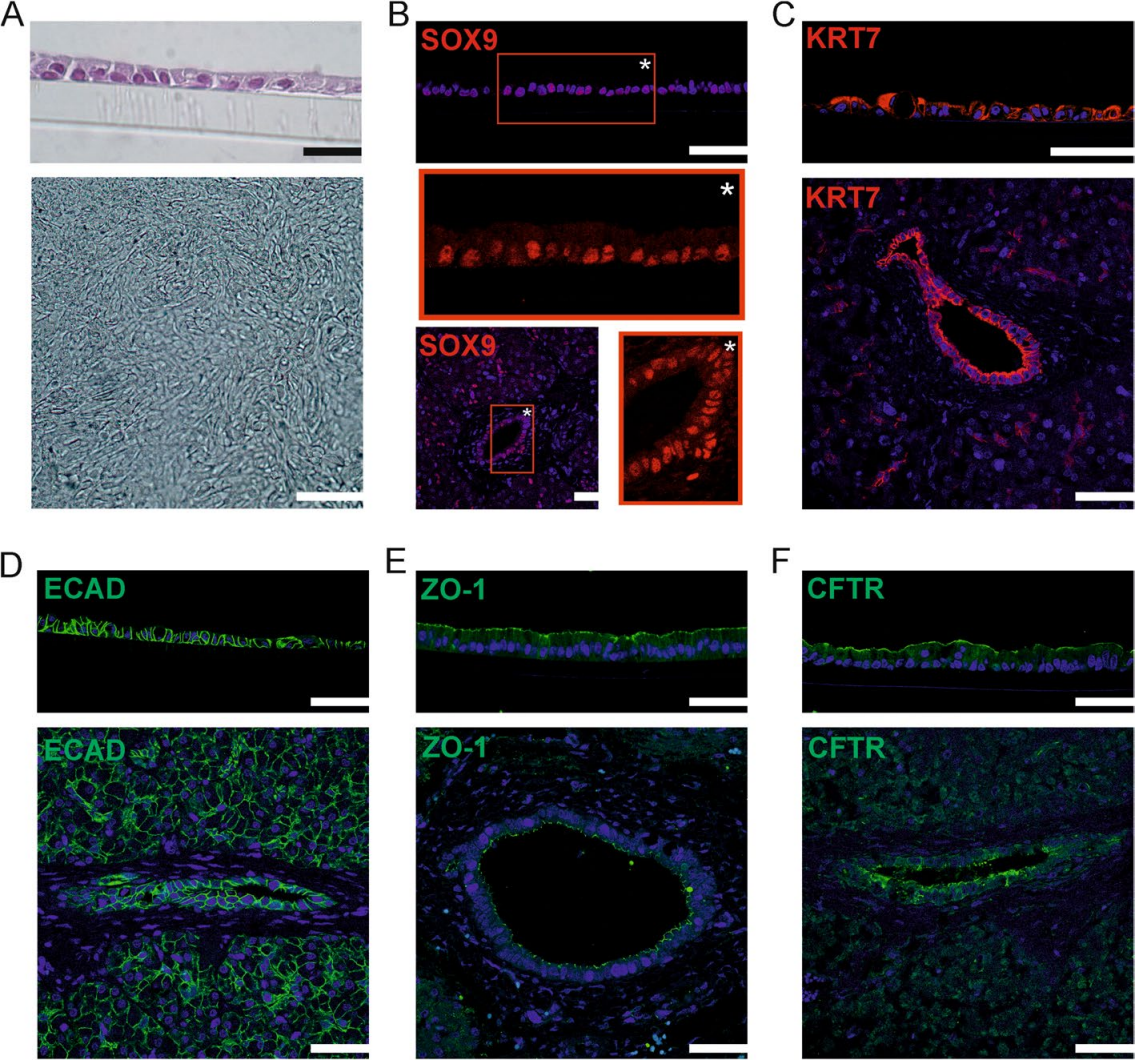
Pancreatic organoid-derived cells form a polarized epithelium when cultured on a permeable substrate. **A** Top panel: transverse section of an epithelial monolayer cultured on a Transwell insert (HE-stain). Scale bar: 25 µm. Bottom panel: the apical aspect of a confluent monolayer (bright-field microscopy). Scale bar: 100 µm. **B** Epithelial monolayers (top) and pancreatic tissue (bottom) were stained for SOX9. Nuclei are visualized using DAPI staining. Insets show SOX9 staining only in the area denoted by an asterisk. Scale bars: 50 µm. **C–F** Epithelial monolayers (top panels) and pancreatic tissue (bottom panels) were stained for KRT7, ECAD, ZO-1 or CFTR. Nuclei are visualized using DAPI staining. Scale bars: 50 µm. Fluorescence was visualized on a Stellaris 5 low incidence angle upright confocal microscope with a HC PL APO CS2 40x/1.30 oil objective (Leica Microsystems)

When cells derived from these ductal organoids were seeded on a permeable substrate they formed confluent monolayers within 1–2 weeks (Fig. 3A). We used immunocytochemistry to characterize these monolayers, and to compare the cellular partitioning of proteins to that of native pancreatic tissue. Cultured cells, as well as ductal epithelial cells in pancreatic tissue, showed nuclear localization of the transcription factor SOX9 (Fig. 3B), and stained positive for the ductal epithelial marker keratin 7 (KRT7; Fig. 3C). The cell-to-cell adhesion molecule e-cadherin (ECAD, *CDH1*) was localized to the lateral plasma membrane of cells in pancreatic tissue and monolayers, suggesting the formation of adherens junctions (Fig. 3D). Zonula occludens 1 (ZO-1; *TJP1*) was localized to the lumen-facing cell surface in ductal tissue, at the junction between adjacent cells, congruent with its function in the formation of tight junction complexes (Fig. 3E). A similar partitioning was observed in monolayers, indicating the formation of a highly polarized epithelium. Crucially, this was also suggested by the localization of CFTR, which was confined to the apical plasma membrane of cells in monolayers (Fig. 3F). In pancreatic tissue, CFTR was localized exclusively to the luminal membrane of the ducts.

### Ductal epithelial monolayers display CFTR- and CaCC-mediated anion secretion

**Fig. 4:**
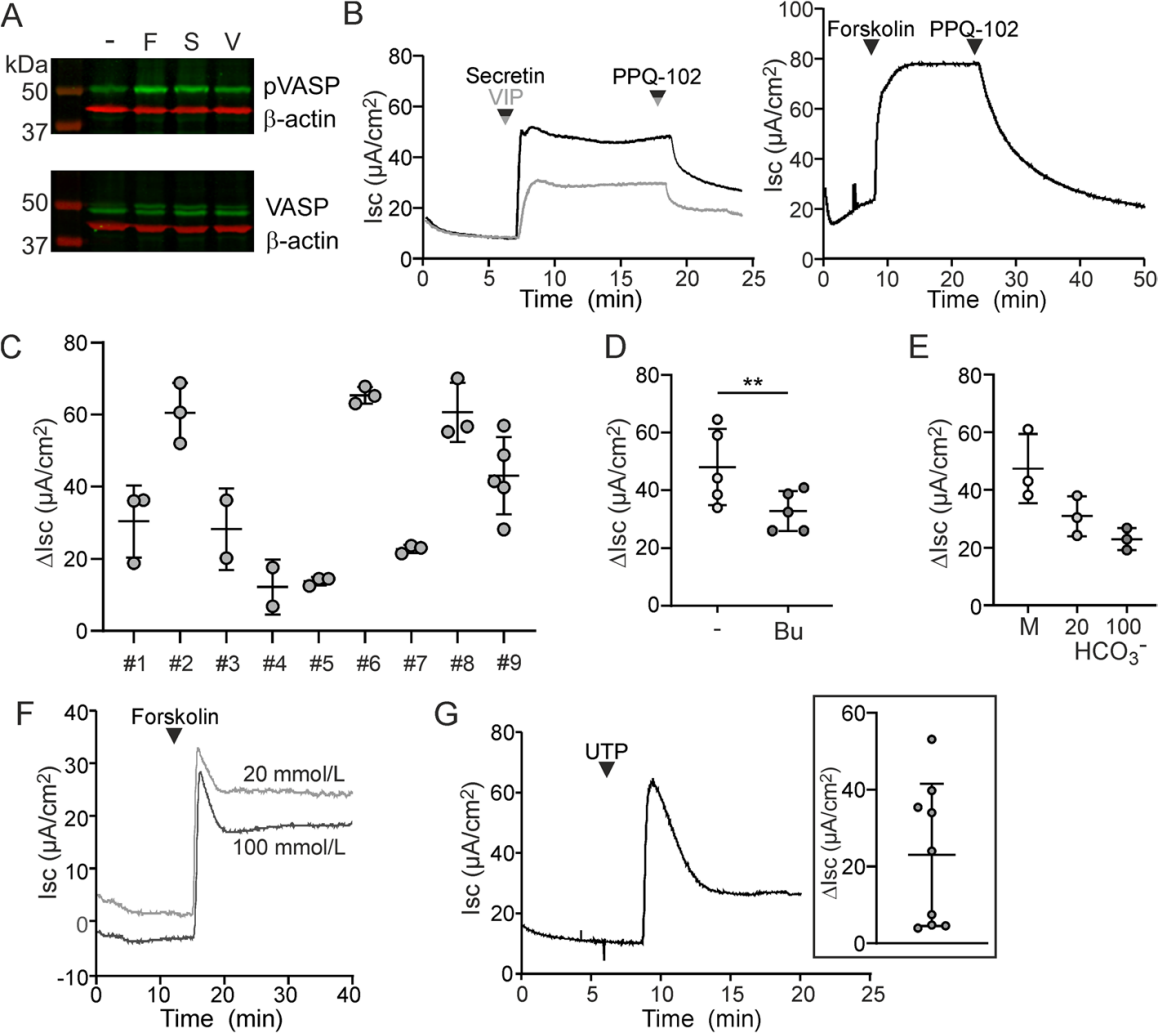
Pancreatic organoid-derived epithelial monolayers recapitulate ductal anion secretory pathways. **A** The effect of forskolin (F), secretin (S) and VIP (V) on PKA-mediated phosphorylation of VASP (top panel) and total VASP (bottom panel) levels was assessed by Western blot analysis. Detection of β-actin served as a loading control. Note that phosphorylation of Ser-157 decreases the electrophoretic mobility of VASP. Outer left lane shows the position of molecular weight markers, with an approximate molecular mass as indicated. **B** Representative experiments showing the effect of secretin, VIP, forskolin and PPQ-102 on the anion secretory current. **C** The cAMP-dependent Isc responses in nine organoid lines obtained from different animals. Each data point represents one technical replicate. **D** Forskolin-dependent Isc responses in the presence or absence of bumetanide (Bu). Each data point represents one technical replicate. Data were obtained from two organoid lines. **P= 0.006. Statistical analysis: paired t-test. **E** Forskolin-dependent Isc responses of monolayers bathed in Meyler solution (M), or in bathing solution containing only bicarbonate as the CFTR-permeating anion. Bicarbonate secretion was assayed at luminal bicarbonate concentrations of 20 or 100 mmol/L, as indicated. Each data point represents one organoid line. **F** Representative experiment demonstrating the forskolin-dependent Isc response at luminal bicarbonate concentrations of 20 or 100 mmol/L. **G** Effect of UTP on the anion secretory current. The inset shows the peak UTP-dependent Isc response, assessed in the presence of PPQ-102, in nine organoid lines

The intestinal hormone secretin and the neuronal signaling peptide VIP are known to control ductal fluid secretion through activation of Gs-coupled receptors that promote the production of cAMP and activation of PKA [39]. To assess whether ductal organoids express functional secretin and/or VIP receptors, we analyzed phosphorylation of the PKA substrate VASP, an adaptor protein that is associated with the cell cytoskeleton [43]. We observed that stimulation of organoids with either secretin, VIP, or the direct adenylyl cyclase agonist forskolin, triggered robust PKA-mediated Ser-157 phosphorylation of VASP (Fig. 4A, Additional file 1: Figs. S2, S3).

Next, we sought to ascertain whether activation of this (neuro)endocrine signaling pathway leads up to an anion secretory response. To this end, we assessed electrogenic ion transport across ductal epithelial monolayers. We observed that secretin, VIP and forskolin acutely triggered anion secretion (Fig. 4B). This response was blocked by the CFTR inhibitor PPQ-102. We consistently observed cAMP- and CFTR-dependent anion secretion in all cultures that were established from different animals. However, the magnitude of the response varied considerably between different preparations (Fig. 4C).

An important function of the ductal epithelium is the secretion of bicarbonate. Bicarbonate secretion may be mediated by CFTR directly, or by the chloride-bicarbonate exchanger PAT1 (*SLC26A6*), which activity may depend on the operation of CFTR as a chloride shunt [38, 39]. To estimate the contribution of bicarbonate secretion to the forskolin-dependent Isc response, we used bumetanide to block NKCC1-dependent chloride secretion. We found that, in the presence of bumetanide, 73 ± 5% (n = 5) of the Isc response was retained (Fig. 4D). Next, we measured the forskolin-dependent Isc response in a bathing solution that contained bicarbonate (20 mmol/L), but no chloride or any other CFTR-permeating anion except bicarbonate. The absence of extracellular chloride excludes any possible contribution of exchangers like PAT1 to bicarbonate secretion. Consistent with the minor effect of bumetanide, we observed that the removal of chloride attenuated the forskolin-dependent Isc response, but that most of the Isc response was retained and must be attributed to bicarbonate secretion (65 ± 4%; n = 3; Fig. 4E). To mimic conditions in the distal part of the ductal tract, we also assessed bicarbonate secretion at a high luminal bicarbonate concentration (100 mmol/L), while keeping the bicarbonate concentration of the contra-luminal bathing solution at 20 mmol/L. We found that, even in the presence of such an unfavorable concentration gradient, the epithelial monolayers secreted bicarbonate when stimulated with forskolin (Fig. 4E, F).

Purinergic receptor activation, through increasing the cellular Ca^2+^ concentration, is known to activate CaCCs in various gastrointestinal epithelia, including that of the pancreatic ductal tree [44]. Presently, we observed that the purinergic receptor agonist UTP, when applied to the luminal bathing solution, elicited an anion secretory response in ductal epithelial monolayers (Fig. 4G). The effects of UTP were assessed in the presence of PPQ-102, indicating that these responses were mediated by an anion channel other than CFTR. The molecular identity of this CaCC is uncertain. A plausible candidate is anoctamin 1/TMEM16A, but based on our transcriptome data and previous reports of the anion channel properties of members of the anoctamin family, we cannot exclude that anoctamin 6/TMEM16F may also contribute to this activity [41, 44].

### Pro-inflammatory cytokines enhance CFTR expression and CFTR-dependent anion transport in ductal epithelial monolayers

**Fig. 5:**
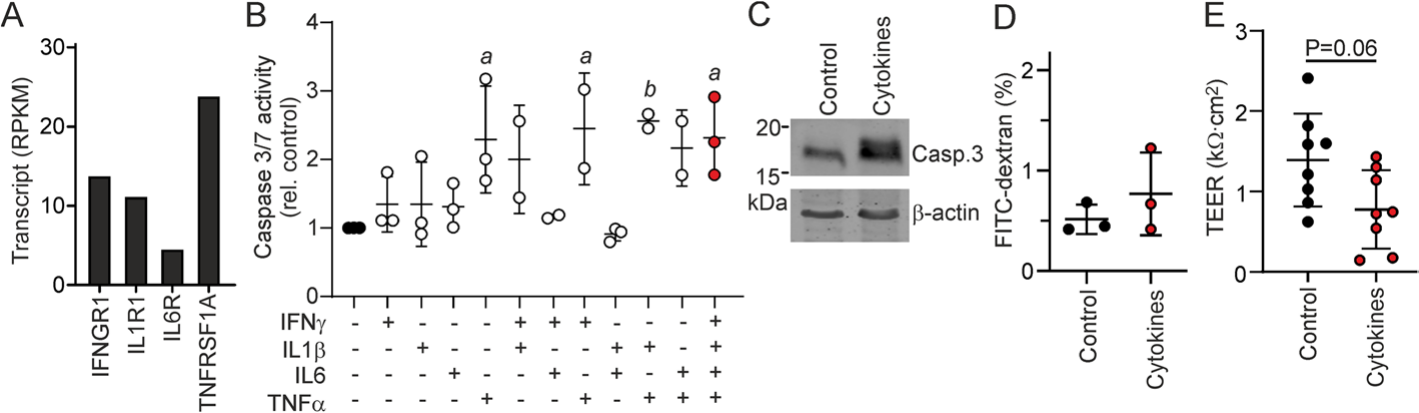
Effects of pro-inflammatory cytokines on apoptotic signaling and epithelial barrier function in pancreatic ductal organoids and epithelial monolayers. **A** Transcript levels of cytokine receptors. **B** Caspase 3/7 activity in epithelial monolayers after 20 h exposure to cytokines. Each data point represents one technical replicate. Statistical analysis: ANOVA. ^*a*^P = 0.04, ^*b*^P = 0.02, compared to untreated. **C** Cleavage of caspase 3 in organoids upon incubation with IL-1β, IL-6, IFN-γ and TNF-α was assessed by Western blot analysis. Detection of β-actin served as a loading control. **D** FITC-dextran transport across epithelial monolayers in the presence or absence of IL-1β, IL-6, IFN-γ and TNF-α. Data represent the FITC-dextran levels in the contra-luminal compartment, relative to the concentration in the luminal compartment at the start of the experiment. Each data point represents one technical replicate. **E** Transepithelial electrical resistance (TEER) of epithelial monolayers mounted in Ussing chambers, in the presence or absence of IL-1β, IL-6, IFN-γ and TNF-α. Each data point represents one technical replicate. Statistical analysis: t-test

Pancreatitis is associated with immune cell infiltration and the release of pro-inflammatory cytokines by antigen-presenting cells of the immune system, upon activation by DAMPs released by injured acinar cells [4]. Typical cytokines produced in this process are IL-1β, IL-6, IFN-γ and TNF-α [3, 6]. Ductal organoids expressed all four receptors of these cytokines (Fig. 5A). As TNF-α is known to trigger cell death under some conditions, we first ascertained whether cytokine exposure stimulates apoptotic activity, and/or affects the integrity of the epithelial barrier [45, 46]. To this end, ductal epithelial monolayers were incubated with cytokines for 20 h, and caspase 3/7 activity and the permeability of the epithelium to fluorescein isothiocyanate (FITC)-dextran was measured. We found that TNF-α alone, or in combination with IL-1β, IL-6, or IFN-γ led to a circa two-fold increase in caspase 3/7 activity (Fig. 5B). Stimulation of caspase 3 cleavage by this combination of cytokines was confirmed by Western blot analysis (Fig. 5C, Additional file 1: Fig. S4). Cytokine treatment did not enhance translocation of FITC-dextran across ductal epithelial monolayers, suggesting that barrier function was not acutely affected by this moderate increase in apoptotic activity (Fig. 5D). Still, we observed that the transepithelial electrical resistance (TEER), which principally reflects the permeability of the epithelium to ions, tended to be lower in cytokine-exposed monolayers (Fig. 5E).

**Fig. 6:**
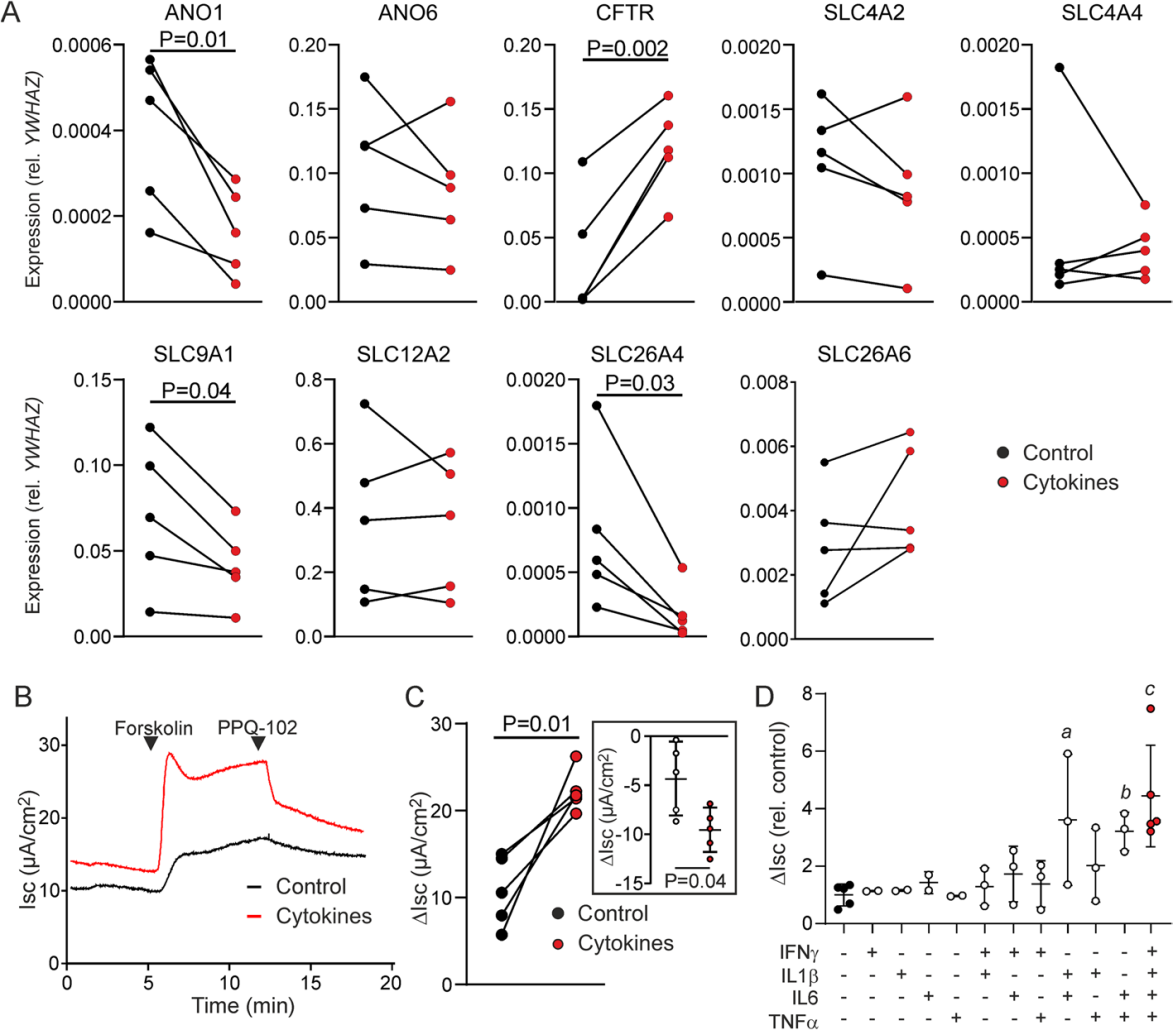
Effect of pro-inflammatory cytokines on anion transport in pancreatic ductal organoid-derived epithelial monolayers. **A** Effect of combined IL-1β, IL-6, IFN-γ and TNF-α exposure (20 h) on the expression of genes involved in ductal anion secretion. **B** Representative experiment showing the effect of combined IL-1β, IL-6, IFN-γ and TNF-α exposure (20 h) on the forskolin-dependent, PPQ-102-sensitive, Isc response. **C** Forskolin-dependent Isc responses in cytokine-treated and control monolayers. The inset shows the change in Isc elicited by PPQ-102. **D** The effect of different cytokines and combinations thereof on the CFTR-mediated Isc response. Experiments were performed on cultures obtained from three animals. Connecting lines indicate paired experiments performed in parallel, on monolayers cultured from the same organoid line. Statistical analysis: paired t-test (**A**, **C**), ANOVA (**D**). ^*a*^P = 0.02, ^*b*^P = 0.007, ^*c*^P < 0.001, compared to untreated

To investigate putative effects of IL-1β, IL-6, IFN-γ and TNF-α on the anion secretory capacity of the ductal epithelium, we assessed the effect of cytokine treatment on the expression of the principle ion transport mechanisms involved in ductal bicarbonate and chloride secretion. Previous studies have shown that these same cytokines, either individually or in combination, and at similar concentrations as applied in our present assays, modulate *CFTR* expression and/or channel activity in intestinal, biliary, and respiratory epithelial cells [22, 23, 25, 27–29]. It has also been shown that pancreatitis is associated with the release of these inflammatory mediators [3–6]. Strikingly, we noted that the combination of these cytokines markedly enhanced expression of *CFTR* (Fig. 6A). In contrast, expression of the chloride-bicarbonate exchanger pendrin (*SLC26A4*), the CaCC anoctamin 1 (ANO1) and the sodium-proton exchanger subtype 1 (*NHE1*) were decreased by cytokine treatment.

In view of the marked induction of *CFTR* by cytokine treatment, we asked whether cytokine treatment may also enhance CFTR-mediated anion secretion across ductal epithelial monolayers. Indeed, treatment of monolayers with a combination of all four cytokines led to a significant increase in the forskolin-dependent Isc response (Fig. 6B, C). Correspondingly, administration of the CFTR blocker PPQ-102 led to a more pronounced decrease in the Isc in cytokine-treated epithelial monolayers than in untreated controls (Fig. 6C). We did not find a similarly robust increase in CFTR activity when we tested the cytokines individually (Fig. 6D). Only IL-6 combined with either IL-1β or TNF-α led to a significant increase in the secretory response, albeit that these combinations failed to mimic the strong effect of the complete cytokine combination. Collectively, these results indicated that stimulation by IL-1β, IL-6, IFN-γ and TNF-α, by transcriptional regulation of the *CFTR* gene, increased CFTR-mediated anion secretion in pancreatic ductal epithelium.

## Discussion

In this study, we characterized pancreatic ductal anion (bicarbonate, chloride) transport and assessed the effect of cytokines typically associated with pancreatitis on ductal CFTR function. The pancreatic ductal epithelial model used in our study was based on the generation of adult stem cell-derived organoids, cultured from ducts of the porcine pancreas [35]. We show that these organoids expressed typical ductal cell markers, including key transport mechanisms involved in transepithelial bicarbonate secretion, such as CFTR, PAT1 (*SLC26A6*), and NBCe1 (*SLC4A4*). In line with earlier work on porcine, bovine and human primary pancreatic ductal cell cultures, we demonstrate that cells derived from these organoids, when seeded on a permeable substrate, formed a highly polarized epithelium [47–50]. When stimulated by either cAMP- (VIP, secretin) or Ca^2+^ (ATP/UTP)-linked neuro-/endo-/paracrine factors, ductal epithelial monolayers displayed anion secretion, congruent with previous studies on isolated native pancreatic ducts [51–53]. Postprandial release of secretin by enteroendocrine cells and VIP by parasympathetic neurons strongly stimulates the bicarbonate and fluid output of the exocrine pancreas [30, 39]. Indeed, we found that only circa a quarter of the current elicited by cAMP/PKA agonists was bumetanide-sensitive (i.e. dependent on NKCC1-mediated chloride uptake), strongly suggesting that most of the response represents bicarbonate secretion.

Although PAT1, which is thought to operate with a 1:2 Cl^−^/HC${\text{O}}_{3}^{ - }$ stoichiometry, may mediate net anion secretion, we conclude that the current elicited by cAMP agonists in the presently studied epithelial monolayers is mediated predominantly or entirely by CFTR [39, 54]. Firstly, because it was sensitive to pharmacological CFTR inhibition. Secondly, because we observed that in the absence of extracellular chloride, which, because their operation is fully dependent on luminal chloride, excludes any involvement of SLC26-type anion exchangers, the monolayers still secreted bicarbonate. Previous work has shown that bicarbonate and fluid secretion across the pancreatic ductal epithelium is highly dependent on CFTR, but the exact role of CFTR in ductal bicarbonate secretion is not fully understood [11, 39]. Ductal cells are thought to secrete bicarbonate principally via Cl^−^/HC${\text{O}}_{3}^{ - }$ exchangers belonging to the SLC26 family, like PAT1 [38, 39]. In this scenario, CFTR may primarily be required to transport chloride, entering the cells via the exchanger, back to the lumen. However, as luminal chloride is replaced by bicarbonate, the driving force for Cl^−^/HC${\text{O}}_{3}^{ - }$ exchange is gradually diminished, and computational modeling predicts that SLC26-type exchangers contribute progressively less to cellular bicarbonate extrusion as the luminal concentration approaches the levels found in the final product [54]. These considerations have led to the assumption that, to reach the final high bicarbonate concentrations observed in human and porcine pancreatic juice, bicarbonate extrusion from epithelial cells, at least in the distal section of the ducts, must principally be channel (i.e. CFTR)-mediated [39, 54]. Consistent with this view, our data demonstrate that bicarbonate secretion across ductal epithelium can occur directly via CFTR, without involvement of PAT1 or any other apically located Cl^−^/HC${\text{O}}_{3}^{ - }$ exchanger. Moreover, we observed robust cAMP-dependent bicarbonate secretion in monolayers that were exposed to luminal bicarbonate concentrations approaching those encountered in the distal section of the ductal tree, demonstrating that ductal epithelial cells can sustain a driving force for bicarbonate efflux, even in the presence of a steep, opposing concentration gradient.

Even though all organoid lines were cultured from ducts isolated from an anatomically defined region of the pancreas, we observed marked differences in the amplitude of the cAMP-dependent anion secretory response between preparations. Each culture was initiated from only a limited amount of ductal fragments, and it is conceivable that separate organoid lines were derived from stem cells originating from different parts of the ductal tree. Immunolocalization and in situ hybridization studies indicate that CFTR can be found in all sections of the pancreatic ductal epithelium, but that its levels vary, and that expression is most prominent in the intralobular and centroacinar cells of the proximal tract [55, 56]. Therefore, we surmise that the variation observed in the secretory response of monolayers, may reflect regional differences in the expression and activity of CFTR in the ductal epithelium. In addition, regional variation in the expression and activity of other key transporters (e.g. NBCe1, NKCC1) may also affect anion secretory capacity. Further, we cannot exclude that other factors, such as genetic variation between individual animals, play a role. In all organoid lines that were established, we also detected UTP-dependent anion secretory responses, consistent with the expression of purinergic P2Y2 receptors. As for CFTR, there is evidence for the presence of CaCC activity in proximal as well as in distal sections of the ductal tree. ATP/UTP-dependent secretory responses have previously been detected in cells isolated from the main (distal) ducts of the bovine pancreas, in mice intra-/interlobular ducts, guinea pig interlobular ducts, and in human pancreatic cell lines [44, 49, 52, 53, 57]. The molecular identity of this CaCC has not been definitely established yet, but ANO1 (TMEM16A) appears to be the most plausible candidate [35, 44, 58].

Pancreatitis is associated with immune cell infiltration and the local, immediate release of pro-inflammatory cytokines [4]. Our results indicate that select cytokines that are key mediators of the pancreatic inflammatory response markedly increased expression of CFTR in ductal epithelium, and stimulated CFTR-mediated ductal anion secretion. Induction of *CFTR* required a combination of pro-inflammatory cytokines, as individual cytokines were ineffective. Similar has been observed for epithelia of the biliary and respiratory tract [23–25, 29]. However, although enhancing *CFTR* expression, the combination of IL-1β, IL-6 and IFN-γ lowered cAMP-dependent anion secretion across bile duct epithelium, indicating cytokines affect the expression or activity of additional ion transport mechanisms or enzymes involved in biliary ductal anion secretion [29]. Stimulatory effects of cytokines on CFTR activity have previously been described for epithelia of the airways, but, to our knowledge, the effect of immune modulators on CFTR function in the pancreatic ductal epithelium has not been investigated. For lung tissue and cell models, it has been shown that a combination of cytokines involved in airway host defense promote CFTR expression and enhance CFTR-mediated bicarbonate secretion [22–24]. By increasing luminal pH, this is thought to improve mucociliary clearance and killing of bacteria, and, consequently, play an important role in the host defense response [25]. We propose that up-regulation of CFTR in the pancreatic ducts by inflammatory mediators may, similarly, serve to accelerate the removal of pathogenic stimuli from the ductal tree, whether endogenous (bile acids, digestive enzymes) or environmental (microbial, chemical) stressors. In this manner, stimulation of CFTR activity could help to contain the progression of pancreatitis at an early stage, and limit tissue injury.

Our data indicate that the pancreas employs a different repertoire of cytokines to promote CFTR activity than the airways [22, 25]. In both organs, the prototypical pro-inflammatory cytokines IL-1β and TNF-α appear to be involved, but the induction of *CFTR* in pancreatic epithelia did not require IL-17, whereas this cytokine was crucial in the response of airway epithelial cells [25]. Conversely, it is unknown whether IL-6, which was essential for inducing *CFTR* in pancreatic ductal cells, affects *CFTR* expression and/or activity in respiratory epithelium. This disparity is probably reflective of the differences between both organs with respect to the specific cytokines involved in their innate and adaptive immunological defense mechanisms [4, 25, 59]. Also, other than in airway epithelium, we found that cytokines did not increase the expression of the chloride-bicarbonate exchangers that potentially contribute to ductal bicarbonate secretion. Whereas expression of the Cl^−^/HC${\text{O}}_{3}^{ - }$ exchanger pendrin (*SLC26A4*) is strongly induced by pro-inflammatory cytokines in respiratory epithelium, in pancreatic cells, expression of *SLC26A4* was suppressed by cytokines and no induction of the principle Cl^−^/HC${\text{O}}_{3}^{ - }$ exchanger expressed in pancreatic ductal epithelium, PAT1 (*SLC26A6*), was observed [24, 25].

No in vitro model can fully emulate the complex immunological environment of pancreatitis in vivo. Consequently, our study has limitations. Firstly, only a select number of cytokines were probed, and we consider it probable that further signaling molecules secreted by immune- or epithelial cells play a role in the acute regulation of CFTR activity and ductal anion secretion. Secondly, we did not take into account the role of direct interactions of ductal epithelial cells with infiltrating immune cells or the involvement of stellate cells, which may have important immune modulatory roles [4, 60]. Chronic inflammation of the pancreas is known to trigger fibrosis and lead to the gradual loss of ductal function, so it is also probable that the effects of prolonged cytokine exposure are different from the more short-term effects investigated here [4, 7]. For instance, we show that TNF-α only moderately stimulated pro-apoptotic signaling, but, upon prolonged exposure, this cytokine has been shown to impair epithelial barrier function [45, 46]. Finally, epithelial monolayers derived from adult stem cell-based organoids may not be fully representative of the ductal epithelium in vivo, even though our experiments indicate that the epithelium formed by these cultured cells shares crucial structural and functional features with native ductal epithelium.

## Conclusions

In conclusion, we show that CFTR activity in pancreatic ductal epithelium is enhanced by exposure to a combination of pro-inflammatory cytokines. Induction of *CFTR* in the initial stages of the inflammatory response, by enhancing the secretory capacity of the ductal epithelium, may serve to alleviate the effects of external stressors, attenuating or preventing further tissue injury. Our data are consistent with studies that point towards a more central role of CFTR dysfunction in the pathophysiology of pancreatitis, which indicate that, for some forms, anion secretory defects in the ductal compartment precede acinar pathology [18, 19, 21, 61]. This concept may inform the development of new treatment modalities for pancreatitis. The discovery of small-molecule therapeutics that restore mutant CFTR function, has transformed the treatment of CF, leading to often marked improvements in clinical outcome. These so-called CFTR modulator drugs improved biomarkers of exocrine pancreas function and may reduce the risk of pancreatitis in CF patients [62, 63]. In addition, modulators were shown to rescue pancreatic function in a non-CF model of pancreatitis, indicating that they may also improve wild type CFTR function [18]. Taken together, these findings suggest that drug therapies directed at improving CFTR function, or ductal anion and fluid secretion in general, may be applicable for management of pancreatitis in a wider population.

### Supplementary Information

**Additional file 1: Figure S1.** Detection of CFTR in pancreatic organoid lines and the human intestinal cell line HT29-CL19A by Western blot analysis. Detection of e-cadherin served as a loading control. **Figure S2**. Detection of phosphorylated VASP (Ser-157) by Western blot analysis. Organoids were treated with forskolin, secretin or VIP. Detection of β-actin served as a loading control. **Figure S3**. Detection of total VASP protein by Western blot analysis. Organoids were treated with forskolin, secretin or VIP. Detection of β-actin served as a loading control. **Figure S4**. Detection of cleaved caspase 3 by Western blot analysis. Organoids were treated with a combination of cytokines (IL-1β, IL-6, IFN-γ and TNF-α). Detection of β-actin served as a loading control.

## Data Availability

Data, analytic methods and study materials will be made available to other researchers on request. Datasets are available through NCBI-GEO repository GSE233579.

## Abbreviations

CaCC: Ca^2+^-dependent chloride channel
PKA: cAMP-dependent protein kinase
CF: Cystic fibrosis
CFTR: Cystic fibrosis transmembrane conductance regulator
DAMPs: Damage-associated molecular patterns
ECAD: E-cadherin
FITC: Fluorescein isothiocyanate
IP_3_: Inositol 1,4,5-triphosphate
IRBIT: Receptors binding protein released with IP_3_
IL-1β: Interleukin-1β
IL-6: Interleukin-6
IL-17: Interleukin 17
IFN-γ: Interferon γ
TEER: Transepithelial electrical resistance
KRT7: Keratin 7
TNF-α: Tumor necrosis factor-α
VIP: Vasoactive intestinal peptide
ZO-1: Zonula occludens 1
