# Supplementary material for: Pro-inflammatory cytokines stimulate CFTR-dependent anion secretion in pancreatic ductal epithelium
Source: Cell Mol Biol Lett. 2024 Jan 23;29:18. doi: 10.1186/s11658-024-00537-1 (PMC10807165; doi:10.1186/s11658-024-00537-1)
Supplement: Supplementary file 1 — Additional file 1: Figure S1. Detection of CFTR in pancreatic organoid lines and the human intestinal cell line HT29-CL19A by Western blot analysis. Detection of e-cadherin served as a loading control. Figure S2. Detection of phosphorylated VASP (Ser-157) by Western blot analysis. Organoids were treated with forskolin, secretin or VIP. Detection of β-actin served as a loading control. Figure S3. Detection of total VASP protein by Western blot analysis. Organoids were treated with forskolin, secretin or VIP. Detection of β-actin served as a loading control. Figure S4. Detection of cleaved caspase 3 by Western blot analysis. Organoids were treated with a combination of cytokines (IL-1β, IL-6, IFN-γ and TNF-α). Detection of β-actin served as a loading control. [file 11658_2024_537_MOESM1_ESM.pdf]

Pro-inflammatory cytokines stimulate CFTR-dependent anion secretion in pancreatic ductal epithelium

Dora Angyal, Tessa A. Groeneweg, Anny Leung, Max Desain, Kalyan Dulla, Hugo R. de Jonge (Deceased), Marcel J.C. Bijvelds

Figure S1

Detection of CFTR in pancreatic organoid lines and the human intestinal cell line HT29-CL19A by Western blot analysis. Detection of e-cadherin served as a loading control.

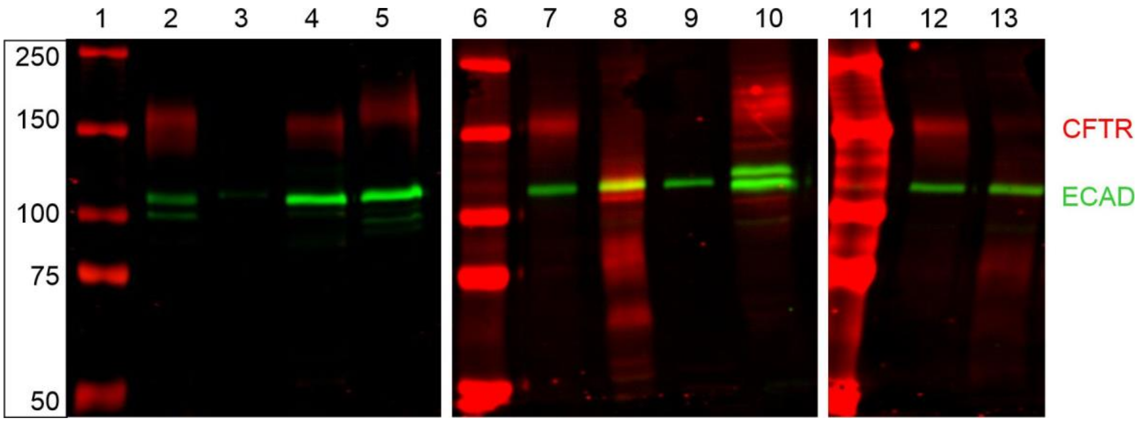

Samples:

1. Markers blot 1; MW as indicated at the left of the blots (kDa).
2. Organoid line #1
3. –
4. Organoid line #2
5. HT29/19A
6. Markers blot 2
7. Organoid line #3
8. Organoid line #4
9. Organoid line #5
10. HT29/19A
11. Markers blot 3
12. Organoid line #6
13. Organoid line #4

Figure S2

Detection of phosphorylated VASP (Ser-157) by Western blot analysis. Organoids were treated with forskolin, secretin or VIP. Detection of β-actin served as a loading control.

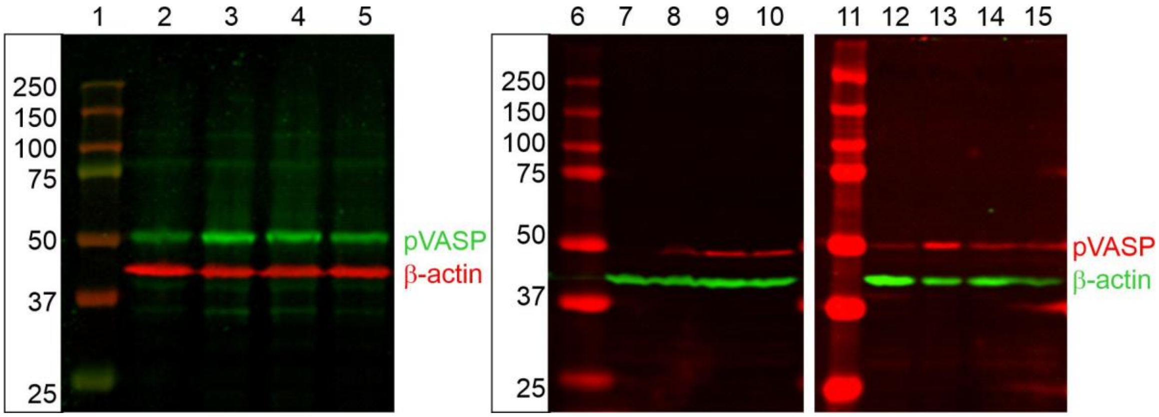

Samples:

1. Markers blot 1; MW as indicated at the left of the blots (kDa).
2. Organoid line #1: Control
3. Organoid line #1: Forskolin
4. Organoid line #1: Secretin
5. Organoid line #1: VIP
6. Markers blot 2
7. Organoid line #2: Control
8. Organoid line #2: Forskolin
9. Organoid line #2 Secretin
10. Organoid line #2: VIP
11. Markers blot 3
12. Organoid line #3: Control
13. Organoid line #3: Forskolin
14. Organoid line #3: Secretin
15. Organoid line #3: VIP

Figure S3

Detection of total VASP protein by Western blot analysis. Organoids were treated with forskolin, secretin or VIP. Detection of β-actin served as a loading control.

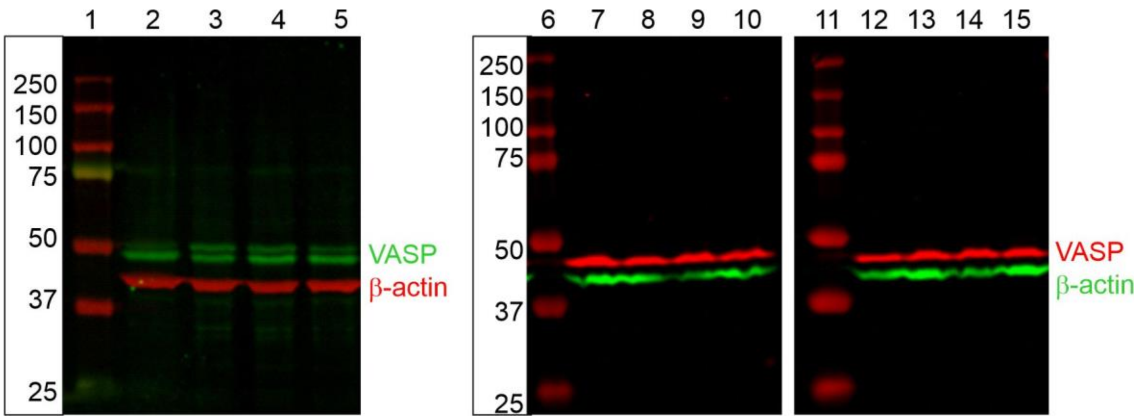

Samples:

1. Markers blot 1; MW as indicated at the left of the blots (kDa).
2. Organoid line #1: Control
3. Organoid line #1: Forskolin
4. Organoid line #1: Secretin
5. Organoid line #1: VIP
6. Markers blot 2
7. Organoid line #2: Control
8. Organoid line #2: Forskolin
9. Organoid line #2 Secretin
10. Organoid line #2: VIP
11. Markers blot 3
12. Organoid line #3: Control
13. Organoid line #3: Forskolin
14. Organoid line #3: Secretin
15. Organoid line #3: VIP

Figure S4

Detection of cleaved caspase 3 by Western blot analysis. Organoids were treated with a combination of cytokines (IL-1β, IL-6, IFN-γ and TNF-α). Detection of β-actin served as a loading control.

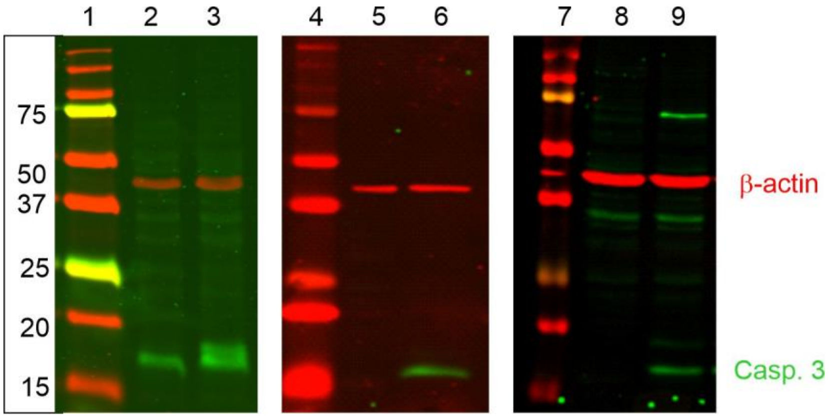

Samples:

1. Markers blot 1; MW as indicated at the left of the blots (k Da).
2. Organoid line #1: Control
3. Organoid line #1: Cytokines
4. Markers blot 2
5. Organoid line #2: Control
6. Organoid line #2: Cytokines
7. Markers blot 3
8. Organoid line #3: Control
9. Organoid line #3: Cytokines
